# Shared and unique alterations of large-scale network connectivity in drug-free adolescent-onset and adult-onset major depressive disorder

**DOI:** 10.1038/s41398-024-02974-0

**Published:** 2024-06-12

**Authors:** Ximan Hou, Rui Liu, Yuan Zhou, Lin Guan, Jingjing Zhou, Jing Liu, Mengqi Liu, Xiaofei Yuan, Yuan Feng, Xu Chen, Aihong Yu

**Affiliations:** 1 Beijing Key Laboratory of Mental Disorders, National Clinical Research Center for Mental Disorders & National Center for Mental Disorders, Beijing Anding Hospital, Capital Medical University, Beijing, China; 2 Advanced Innovation Center for Human Brain Protection, Capital Medical University, Beijing, China; 3 Department of Radiology, Beijing Anding Hospital, Capital Medical University, Beijing, China; 4 CAS Key Laboratory of Behavioral Science, Institute of Psychology, Beijing, China; 5 Department of Psychology, University of Chinese Academy of Sciences, Beijing, China

**Keywords:** Depression, Diagnostic markers

## Abstract

Differences in clinical manifestations and biological underpinnings between Major Depressive Disorder (MDD) onset during adolescence and adulthood have been posited in previous studies, implying an influential role of age of onset (AOO) in the clinical subtyping and therapeutic approaches to MDD. However, direct comparisons between the two cohorts and their age-matched controls have been lacking in extant investigations. In this investigation, 156 volunteers participated, comprising 46 adolescents with MDD (adolescent-onset group), 35 adults with MDD (adult-onset group), 19 healthy adolescents, and 56 healthy adults. Resting-state functional MRI scans were undergone by all participants. Large-scale network analyses were applied. Subsequently, a 2 × 2 ANOVA was employed to analyze the main effects of diagnosis, age, and their interaction effect on functional connectivity (FC). Furthermore, regression analysis was employed to scrutinize the association between anomalous FC and HAMD sub-scores. Increased FC in visual network (VN), limbic network (LN), VN-dorsal attention network (DAN), VN-LN, and LN-Default Mode (DMN) was found in both adolescent-onset and adult-onset MDD; however, the increased FC in DAN and LN were only found in adult-onset MDD and the decreased FC in DAN was only found in adolescent-onset MDD. Additionally, the relationship between HAMD factor 1 anxiety somatization and altered FC of DAN, VN, and VN-DAN was moderated by AOO. In conclusion, shared and distinctive large-scale network alterations in adolescent-onset and adult-onset MDD patients were suggested by our findings, providing valuable contributions towards refining clinical subtyping and treatment approaches for MDD.

## Introduction

Depression presents a significant public health challenge, with a high occurrence and disability rate, placing a considerable burden on families and society [1]. Its prevalence peaks during adolescence, affecting around 25.2% of individuals transitioning from childhood to adulthood [2, 3]. Studies indicate that adolescent-onset depression (<18 years) is linked to a heightened risk of mania, more severe symptoms, psychotic manifestations, and an increased likelihood of suicide compared to adult-onset depression (≥18 years) [4, 5]. Moreover, adolescent-onset depression tends to involve enduring depressive episodes and the use of mood stabilizers [4]. Apart from differences in clinical symptoms and course, research also points to disparities in neural mechanisms, including brain functions and structures, between adolescent-onset and adult-onset depression. Consequently, there is a debate among researchers regarding whether adolescent-onset depression and adult-onset depression should be regarded as distinct subtypes of Major Depressive Disorder (MDD) [6–8]. Some argue that these disparities stem from the chronic recurrence of MDD, while others suggest genuine differences between the two age-of-onset (AOO) categories [9, 10]. Therefore, investigating the neurophysiological mechanisms of adolescent-onset and adult-onset depression through neuroimaging is crucial for advancing clinical diagnosis and treatment strategies.

In recent years, the utilization of resting-state functional magnetic resonance imaging (rs-fMRI) has unveiled various brain networks within the human brain [11]. Studies indicate that these networks undergo maturation from adolescence to adulthood, displaying differential functional connectivity (FC) within and between networks in adolescents compared to adults [12]. Mental disorders like depression can disrupt the typical trajectory of brain development [13, 14], leading to numerous investigations into alterations in brain networks among both adolescent and adult populations affected by depression. However, existing studies on MDD brain networks have primarily concentrated on a single age group.

Consistent findings of large-scale brain network dysfunction have surfaced in adults with depression. A meta-analysis spotlighted reduced FC in the frontoparietal network (FPN) and FPN-dorsal attention network (DAN), alongside increased FC in the default mode network (DMN) and DMN-FPN among MDD patients compared to healthy controls (HCs) [15]. Another review pinpointed increased FC within the anterior DMN, heightened FC between the anterior DMN and salience network (SN), and decreased FC between the posterior DMN and central executive network (CEN) in MDD patients relative to HCs [16]. Furthermore, a recent review indicated elevated functional connectivity between the DMN and FPN, and reduced connectivity between the limbic network (LN) and SN in MDD patients compared to HCs [17]. However, these studies involving adult MDD patients might encompass individuals with adolescent-onset depression, overlooking the potential impact of AOO on large-scale brain network alterations, potentially contributing to inconsistent research findings. Researchers have also delved into structural and functional connectivity distinctions in the brains of individuals with adolescent-onset and adult-onset MDD by investigating adult patients. Evidence suggests that the onset age of depression correlates with anomalies in the emotion-regulating network, suggesting the possible classification of early-onset depression as a distinct subtype [18]. Nevertheless, such inquiries struggle to disentangle the effects of early-onset from those associated with recurrent episodes on the brain’s developmental processes. Consequently, the shared and unique impacts of adolescent-onset MDD (in adolescents) and adult-onset MDD on brain function remain ambiguous.

Several studies have looked into the malfunction of large-scale brain networks in adolescents with depression. Sacchet et al. identified decreased connectivity within parts of the attention, central executive, salience, and default mode networks in adolescent patients with MDD compared to appropriately matched healthy controls. Importantly, the reduced connectivity of the DAN showed a significant correlation with the duration of depression [14]. Similarly, Pan et al. noticed lower connectivity of the cognitive control network (CCN) in depressed adolescents compared to matched healthy controls, and this decreased connectivity was associated with disease severity and executive dysfunction [19]. Other research revealed diminished FC within the limbic and salience networks in treatment-naive MDD adolescents compared to healthy controls [20]. Taken together, these studies suggest a prevalence of decreased FC across various large-scale networks in adolescents with MDD. Considering these findings alongside those regarding adult depressive patients, it is conceivable that shared and distinct alterations in FC exist between adolescent MDD patients and those with adult-onset MDD. However, limited research has directly compared these two patient groups.

A single study has examined differences in large-scale brain networks between adolescent and adult MDD. A recent multi-center study, involving a large sample of 617 patients with depression and 621 healthy individuals, aimed to clarify common and distinct functional changes in brain networks across adolescents, middle-aged, and elderly individuals with MDD [21]. This study revealed an interaction effect between diagnosis and age on FC within DMN. Furthermore, it observed increased connectivity within the sensorimotor-subcortical network among adolescent patients, decreased connectivity within the visual-subcortical network among early- to middle-aged adults, and decreased connectivity within various networks, including the subcortical, default-mode, cingulo-opercular, and attention networks, among late adults compared to healthy controls [21]. However, it’s important to note that this study might have included a mixture of patients with adolescent-onset and adult-onset MDD within the adult MDD group. Additionally, the study did not control for the impact of medication use, which could introduce an additional confounding factor into the results.

In this current study, our aim is to investigate shared and unique changes in large-scale network connectivity between two different AOO levels of MDD. We hypothesize that both shared and distinct alterations in FC exist between patients with adolescent-onset MDD and those with adult-onset MDD, with generally lower FC observed in adolescent-onset MDD compared to healthy controls, and either higher or lower FC in adult-onset MDD compared to healthy controls. To achieve this, we recruited drug-free adolescent patients with MDD and adult patients with adult-onset MDD, thus removing the influence of medication and AOO on large-scale brain networks. Specifically, we performed a comparative analysis of FC in large-scale brain networks between drug-free adolescent-onset MDD and adult-onset MDD using a 2×2 ANOVA, with age and diagnosis as independent variables. Additionally, we examined the relationship between abnormal patterns of FC and clinical symptoms.

## Materials and methods

### Participants

A total of 156 volunteers participated in the study. This group comprised 46 adolescents with MDD (referred to as the adolescent-onset group), 35 adults with MDD (referred to as the adult-onset group), 19 healthy adolescents, and 56 healthy adults. Participants were recruited from Beijing Anding Hospital, Capital Medical University. Criteria for inclusion in the MDD groups were: (1) adolescents aged 12–18 with MDD onset during the same age range, and adults aged 25–50 with MDD onset during the same age range; (2) of Han ethnicity and right-handed; (3) meeting the Diagnostic and Statistical Manual of Mental Disorders-IV (DSM-IV) criteria for MDD; (4) not taking antidepressant medications or receiving them for no more than seven days within the preceding fourteen days before enrollment. Exclusion criteria for MDD participants were: (1) having a significant DSM-IV diagnosis other than depression; (2) a history of alcohol dependence; (3) pregnancy or breastfeeding; (4) contraindications for MRI scanning; (5) presence of psychotic symptoms. Criteria for inclusion in the healthy control (HC) group were: (1) adolescents aged 12–18 and adults aged 25–50; (2) of Han ethnicity and right-handed; (3) no history of mental illness; (4) absence of major chronic diseases. Exclusion criteria for HCs were: (1) a family history of mental illness; (2) a history of alcohol dependence; (3) pregnancy or breastfeeding; (4) contraindications for MRI scanning.

The study procedures were approved by the Human Research and Ethics Committee of Beijing Anding Hospital, Capital Medical University (2017-24, (2022) Research No. (70)). In accordance with the latest version of the Declaration of Helsinki, informed consent was obtained from all adult participants and guardians of adolescent participants. All methods were performed in accordance with the relevant guidelines and regulations.

### Clinical assessments

All participants were evaluated using the 17-item Hamilton Rating Scale for Depression (HAMD-17) [22], conducted by the psychiatrist. The HAMD-17 comprises sub-scores for anxiety/somatization, weight, cognitive impairment, retardation, and sleep disturbance [23]. The breakdown of each HAMD-17 sub-score is provided in the supplementary methods.

### Image acquisition

Structural and functional MRI scans were performed using a German Siemens Prisma 3.0 T superconducting MR Scanner equipped with a 64-channel head coil. Earplugs were given to participants to reduce noise, and they were positioned supine with their heads facing forward. A sagittal T1-weighted magnetization-prepared rapidly acquired gradient-echo (MPRAGE) sequence and a gradient-recall echo-planar imaging (EPI) sequence were utilized. Participants were instructed to keep their heads still throughout the scan. Scan parameters were set as follows: (1) T1-MPRAGE sequence: repetition time (TR) = 2530 ms, echo time (TE) = 1.85 ms, flip angle (FA) = 15°, matrix = 256 × 256, slice thickness = 1 mm, gap = 0 mm, number of slices = 192, field of view (FOV) = 256 mm × 256 mm. (2) EPI sequence: TR = 2000 ms, TE = 30 ms, FA = 90°, matrix = 64 × 64, slice thickness = 3.5 mm, gap = 0.7 mm, number of slices = 33, FOV = 200 mm × 200 mm. During the rs-fMRI scan, participants were instructed to close their eyes and maintain an awake, relaxed, and calm state. Head movement was minimized with a sponge pad, and earplugs were provided to reduce perceived noise. The duration of the rs-fMRI scan was 6 min and 40 s, during which 200 time points were collected.

### Data preprocessing

A surface-based package was employed for MRI data preprocessing and brain network construction, which is deemed to be more accurate for data processing and investigation of brain function [24]. Surface-based MRI image preprocessing was carried out using DPABISurf_V1.2, a MATLAB-based fMRI brain image analysis software that facilitates surface-based preprocessing. DPABISurf, an extension of DPABI/DPARSF [24], was developed by the Institute of Psychology of the Chinese Academy of Sciences and interfaces with the FMRIPprep docker, integrating FreeSurfer, ANTs, FSL, and AFNI for MRI data preprocessing. Further details on MRI data processing are available in our previous research [25]. Initially, EPI and T1 DICOM data were converted to BIDS format, with the first 10 time points of the EPI data being discarded to eliminate magnetic field instability effects. A reference volume and its skull-stripped version for the rs-fMRI data were generated by fMRIPrep. Subsequently, bbregister was employed to co-register the T1w reference and the BOLD reference based on boundary-based registration. Slice timing correction was conducted using 3dTshift from AFNI. Image data were resampled to two standard spaces: fsaverage5 space for the cortex and MNI152nLin2009cAsym space for the brain stem, cerebellum, and subcortical nucleus. Preprocessing steps included: resampling to 2 mm voxel size, slice timing correction, regression of nuisance covariates (main components of white matter and cerebrospinal fluid signals, and head motion estimation using the Friston 24 model), normalization to standard space (MNI152nLin2009cAsym), surface-based smoothing with a full width at half maximum (FWHM) of 6 mm, and filtering with a frequency range of 0.01–0.1 Hz. Notably, whole-brain signal regression was omitted in our study as the whole-brain signal may contain informative content [26].

### Network construction

Schaefer’s functional Atlas delineated 400 Regions of Interest (ROIs) throughout the brain [11]. Subsequently, seven cortical networks were delineated by aligning them with Yeo’s seven networks parcellation [27]. These seven networks included the sensorimotor network (SMN), ventral attention network (VAN), DAN, visual network (VN), FPN, DMN, and LN. Detailed information regarding the ROIs for the seven networks can be found in Supplementary Table S1.

We computed the mean time series for each brain region in individual participants and calculated Pearson correlation coefficients between any two brain regions to construct a 400 × 400 whole-brain functional connectivity matrix. The resulting matrix was then transformed into z-scores using Fisher’s r-to-z transformation. Based on these connectivity matrices, 7 intra-network functional connectivity (FC) and 21 inter-network FC values were computed for each participant.

### Intra-network connectivity analysis

Intra-network connectivity was assessed by computing the average connectivity strength of all connections within a specific network and normalizing the network connectivity by the square of the number of nodes in the network [28]. A 2 × 2 ANOVA was utilized to identify main effects of diagnosis (MDD vs HC) and age (adolescents vs adults), as well as interaction effects between these two factors on intra-network FC. Gender and mean Framewise Displacement (FD) were included as covariates. Multiple comparison correction was applied using the False Discovery Rate (FDR) correction (*P* < 0.05) [29]. In case of significant effects, post-hoc t-tests or simple effect analyses were conducted (*P* < 0.05, Bonferroni-corrected). These statistical analyses were conducted using R version 4.2.2 with the “anova_test” function from the “rstatix” package.

### Inter-network connectivity analysis

Inter-network connectivity was assessed by computing the average FC strength of all connections between any pair of regions located in two networks, normalized by the product of the number of nodes within each of the two networks [30]. Similar to intra-network FC, a 2 × 2 ANOVA was employed to detect main effects of diagnosis and age, as well as interaction effects on inter-network FC. Gender and mean FD were considered as covariates. Multiple comparison correction was applied using the FDR correction (*P* < 0.05), followed by post-hoc *t* tests or simple effect analyses (*P* < 0.05, Bonferroni-corrected). These statistical analyses were conducted using R version 4.2.2.

Additionally, a sensitive analysis was conducted to examine whether the FC difference was attributable to the duration and the number of episodes. A 2 × 2 ANOVA was performed to detect main effects of diagnosis and age, as well as interaction effects on both inter-network and intra-network FC. Alongside gender and mean FD, the number of episodes and the duration were included as covariates. The results obtained with the regression of the number of episodes or the duration were provided in the supplementary materials.

### Network-based statistic analysis

The Network-Based Statistic (NBS) toolbox was utilized to examine the interaction effect between diagnosis and age on the FC of large-scale brain networks, while addressing the issue of multiple comparisons [31]. A two-way ANOVA test was conducted to identify main effects of diagnosis and age, as well as interaction effects on FC. Gender and FD were accounted for as covariates. In the NBS analysis, the F statistics threshold between any nodes was set at 3.1, and the zero distribution was established through permutation testing with 5000 permutations. The *P* value was computed based on the zero distribution. NBS extends the cluster-level correction principle to the connectivity matrix field, enhancing statistical power by evaluating the properties of the entire graph [31].

### Statistical analysis

Statistical analyses were conducted using SPSS Statistics 26.0 and R version 4.2.2. Two-sample *t* tests, Mann–Whitney *U* tests, or chi-square tests were employed to analyze demographic and clinical characteristics. Chi-squared tests were used to assess gender differences among the four groups. The Kruskal–Wallis test was applied to compare mean FD-power differences among the four groups. Regression analyses were employed to evaluate the relationship between abnormal intra-network or inter-network FC and HAMD-17 sub-scores exhibiting significant statistical differences between the two different AOO patient groups, with gender and mean FD as nuisance covariates. In the presence of an interaction effect, MODPROBE was utilized for simple effect analysis. MODPROBE serves as a tool for probing single-degree-of-freedom interactions in Ordinary Least Squares (OLS) and logistic regression within SPSS [32].

## Results

### Demographic and clinical characteristics

Demographic and clinical information were summarized in Table 1. No significant differences were observed among all participants in gender and FD (all *P* ≥ 0.05). Similarly, no significant differences were found in age and educational attainments within adolescents or adults. Furthermore, no significant difference in HAMD-17 total score was found between the adolescent-onset and adult-onset MDD groups. However, compared to the adult-onset patients, HAMD-17 factor 1 (anxiety/somatization) and HAMD-17 factor 4 (retardation) were higher in adolescent-onset patients (*P* = 0.006, *P* < 0.001). Besides, compared to the adolescent-onset patients, the duration was longer, and the number of episodes was higher in adult-onset patients (*P* = 0.025; *P* = 0.002).

**Table 1:** Demographic of participants.

	Adolescents Adults				*P* value (two-tailed)
	MDD	HC	MDD	HC	
Age (median, range)	15.0 (12–17)	14.0 (12–17)	29.0 (25–46)	28.0 (25–47)	0.489/0.257^b^
Sex (male/female)	10/36	10/9	11/24	24/32	0.050
Education level^a^	34/12	14/5	7/20/8	6/24/26	0.771/0.07^c^
HAMD-17	21.33 ± 5.10	–	21.49 ± 3.57	–	0.875
HAMD-factor 1	5.85 ± 2.05	–	4.63 ± 1.78	–	0.006
HAMD-factor 2	0.39 ± 0.80	–	0.63 ± 0.88	–	0.129
HAMD-factor 3	4.96 ± 2.11	–	3.94 ± 1.61	–	0.011
HAMD-factor 4	12.02 ± 4.60	–	6.86 ± 1.44	–	0.000
HAMD-factor 5	2.57 ± 1.91	–	2.94 ± 1.51	–	0.291
The number of episodes	1.13 ± 0.34	–	1.74 ± 1.60	–	0.002
the duration (month)	16.67 ± 15.40	–	48.60 ± 72.84	–	0.025
FD power	0.12 ± 0.06	0.13 ± 0.05	0.11 ± 0.05	0.11 ± 0.04	0.454
(mean ± 3 s.t.d, mm)					

^a^Education level of adolescents was divided into two categories: junior high school/senior high school: education of adult groups was divided into three categories: junior and senior high school/bachelor’s degree/master’s degree and above.

^b^*P* value of Mann–Whitney test in adolescent groups/*P* value of Mann–Whitney test in adult groups.

^c^*P* value of Chi-square test in adolescent groups/*P* value of Chi-square test in adult groups.

Among adult-onset MDD patients, two individuals took escitalopram for less than 7 days during the current depressive episode, with a half-life of ~30 h. In addition, one patient used oxazepam for 2 days, and another patient took lorazepam for 3 days during the current depressive episode. None of the adult-onset MDD patients used mood stabilizers for nearly 14 days during the current depressive episode. On the other hand, all adolescent-onset MDD patients refrained from using antidepressants, benzodiazepines, or mood stabilizers for nearly 14 days during the current depressive episode.

### Intra-network connectivity analysis

**Fig. 1:**
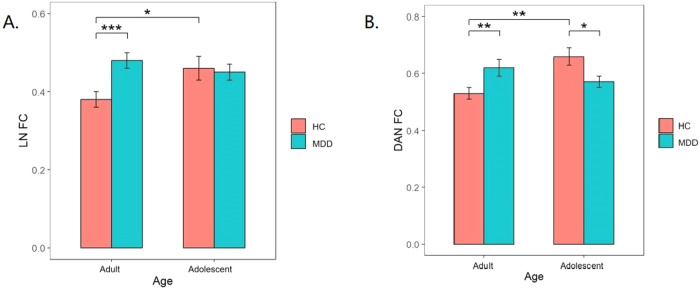
Diagnosis×age interaction. Bar graphs illustrating post-hoc analysis of the interaction effect in DAN connectivity (**A**) and LN connectivity (**B**). *P < 0.05, **P < 0.01, ***P < 0.001. DAN dorsal attention network, LN limbic network.

**Fig. 2:**
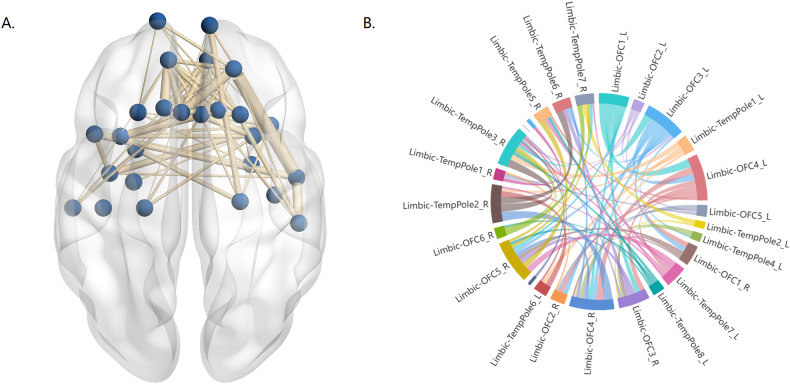
Results of the NBS. An interaction effect between diagnosis and age on FC within components of LN. **A** Axial views from superior to brain where line thickness reflects the effect size for the relevant test. **B** Connectivity diagram where line thickness reflects connectivity strength. NBS Network-Based Statistic, LN limbic network, OFC orbitofrontal cortex.

Supplementary Fig. S1 illustrates the averaged FC matrix among 400 Regions of Interest (ROIs) across the brain in all four groups of participants. We observed a significant main effect of diagnosis on the intra-network FC of VN and LN (*F* = 6.260, pFDR = 0.046; *F* = 12.272, pFDR = 0.004; Fig. 1), indicating that these FC abnormalities were independent of age stratification. Post hoc analysis revealed shared increased network connectivity in the MDD group. Furthermore, we detected an interaction effect between diagnosis and age on the intra-network FC within the DAN and LN (*F* = 10.116, pFDR = 0.013; *F* = 6.550, pFDR = 0.040; Fig. 2). Post-hoc analysis demonstrated that intra-network connectivity of DAN and LN (*P* = 0.008, *P* < 0.001, Bonferroni-corrected) in adult-onset MDD patients was significantly higher than that in the adult HCs, while DAN connectivity in adolescent-onset MDD patients was significantly lower than that in the adolescent HCs (*P* = 0.032, Bonferroni-corrected). Additionally, intra-network connectivity of DAN and LN in adolescent HCs was significantly higher than that in the adult HCs (*P* = 0.002, *P* = 0.011, Bonferroni-corrected). No significant differences were found between the components of other intra-networks. Furthermore, no significant main effect of age was identified among the components of intra-networks.

### Inter-network connectivity analysis

We observed a significant main effect of diagnosis on the inter-network FC of VN-DAN, VN-LN, and LN-DMN (*F* = 8.215, pFDR = 0.048; *F* = 9.624, pFDR = 0.048; *F* = 7.498, pFDR = 0.048; Fig. 1), which was not influenced by age stratification. Post-hoc analysis revealed shared increased network connectivity in the MDD group. No significant interaction effect was found between the connectivity of other networks. Additionally, no significant main effect of age was identified in the connectivity of networks.

### Results of the NBS

To investigate intra-networks with an interaction effect of age and diagnosis, we utilized the NBS method (Supplementary Table S1). NBS analysis specifically targeted the DAN and LN intra-networks, uncovering an interaction effect of age and diagnosis. Within the LN network, NBS identified a significant interaction effect between diagnosis and age on FC (*P* = 0.0478; Fig. 2). The statistically significant edges and nodes numbered 64 and 25, respectively. These significant FC were distributed widely across both cerebral hemispheres, involving regions such as the limbic system, temporal pole, and orbitofrontal gyrus. However, NBS analysis did not reveal a significant interaction effect between diagnosis and age on intra-network FC within the DAN.

### Regression analysis

**Fig. 3:**
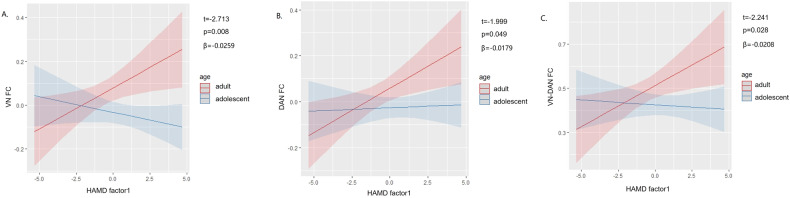
Simple effect analysis. Simple effect analysis of the interaction effect between age and HAMD factor 1 (anxiety somatization) on the FC of VN (**A**), DAN (**B**), and VN-DAN (**C**). HAMD Hamilton Depressive Rating Scale, DAN dorsal attention network, VN visual network.

A significant interaction effect was observed between age and HAMD factor 1 (anxiety somatization) on the FC of VN, DAN, and VN-DAN (*F* = 4.764, *P* = 0.008; *F* = 4.745, *P* = 0.049; *F* = 5.752, *P* = 0.028; Fig. 3). Simple effect analysis revealed a positive correlation between the strength of FC in VN, DAN, and VN-DAN and anxiety somatization scores for adult patients (*β* = 0.038, *t* = 2.402, *P* = 0.019; *β* = 0.039, *t* = 2.637, *P* = 0.010; *β* = 0.037, *t* = 2.455, *P* = 0.016), but no correlation was observed for the adolescent group. No association was found between HAMD factor 4 (retardation) and altered FC.

## Discussion

This research investigated significant network connectivity issues in drug-free patients with MDD, distinguishing between cases with onset in adulthood and adolescence. Our findings revealed both shared and unique network changes in both adolescent and adult-onset MDD. Shared alterations suggested that these effects might be related to depression, regardless of AOO. Unique changes implied that AOO could be specifically responsible for these effects. Our results confirmed adolescence as a critical period in the brain development of MDD, providing insights from neuroimaging to enhance clinical categorization and treatment approaches. These findings contribute to a better understanding of the diversity in MDD onset ages in terms of brain dysfunction.

For the unique alterations, we noticed higher connectivity within the DAN and LN networks in healthy adolescents compared to healthy adults. Typically, short-distance connections decrease as individuals mature, which is consistent with our findings [33–35]. In patients, we found distinct patterns of connectivity within the DAN and LN networks specific to the disease. The DAN network is involved in directing attention toward goals, aiding in the processing of visual stimuli related to tasks that require focused attention [36]. In our study, we observed lower DAN connectivity in adolescents with MDD compared to healthy adolescents, which is in line with previous research [14]. This previous study suggested a potential delay in the development of goal-directed attention in depressed adolescents due to reduced DAN connectivity [14]. Additionally, we found that DAN connectivity was higher in adults with MDD compared to healthy adults, indicating abnormalities in attentional processes [36, 37]. Currently, results regarding DAN intra-network connectivity in MDD adults are inconsistent, possibly explained by the considerable heterogeneity among patients with depression [38]. Some studies showed reduced DAN connectivity in adults with first-episode depression but increased connectivity in those with recurrent depression compared to healthy controls [39]. Another study found decreased connectivity within the DAN network in adults with an anhedonia-dominated subtype of MDD [40]. In our study, the inclusion of adult-onset depression patients without strict control over subtypes and recurrence history, along with a limited sample size, may explain the inconsistency with previous findings. The LN network is essential for emotional memory and is associated with abnormalities in bottom-up processes [41]. We also observed increased connectivity within the LN network in adult-onset MDD patients compared to healthy adults, indicating abnormalities in emotional processing. NBS analysis revealed interaction effects within the LN network. In contrast, previous studies reported decreased connectivity within LN networks in adults with MDD [25]. This disparity could be due to uncontrolled factors such as AOO and medication in previous studies, where adult MDD patients might include both adolescent-onset and adult-onset cases. The distinct patterns of abnormal connectivity moderated by AOO suggest that depression influences the development of normal brain networks differently. These findings suggest that adolescent-onset and adult-onset depression could be considered distinct subtypes, emphasizing the importance of age when exploring biomarkers for MDD.

Regarding the common changes, our study highlighted increased FC in the VN, LN, VN-DAN, VN-LN, and LN-DMN networks in both adolescent-onset and adult-onset MDD groups. Consistent with previous research, these findings indicate abnormalities in both bottom-up and top-down connectivity in depression, regardless of AOO [40, 41]. Processing stimuli involves a balance between bottom-up and top-down mechanisms, and excessive activity in top-down regions may be linked to depression [42]. Bottom-up processing involves rapid, instinctive reactions to environmental stimuli, while top-down processing is slower and relies on prior knowledge and intentional planning [42, 43]. The VN network is responsible for visual processing and assessing the external environment [27]. The increased FC within the VN network suggests abnormal enhancement of visual attention. Similarly, heightened FC within the LN network indicates hyperactivity in the limbic system in response to negative emotional stimuli, leading to an increased response to negative information [44]. The hyperconnectivity in the VN-DAN network may reflect abnormalities in top-down attention processes and enhanced visual perception. Similarly, increased connectivity within the VN-LN network suggests enhanced top-down emotional responses to visual stimuli. The DMN is associated with internally focused thinking [16]. Therefore, heightened connectivity between LN and DMN networks might reflect increased transition from emotional processing to self-referential thinking. Contrary to our hypothesis, we only observed higher, not lower, FC in adult-onset MDD compared to HC, and similarly, we found higher FC in adolescent-onset MDD compared to HC. This could be due to the considerable heterogeneity among MDD patients and the limited sample size.

Moreover, our study highlighted a positive correlation between the FC of DAN, VN, and VN-DAN networks with anxiety somatization scores in adult patients, while no such correlation was observed in the adolescent group, suggesting a disparity in this association between adolescent and adult patients. The disruption in bottom-up mechanisms in adult-onset MDD patients might explain the correlation with anxiety measured by the HAMD Factor 1. During periods of anxiety, the balance between top-down and bottom-up mechanisms is disrupted, leading to dominance of bottom-up processing without adequate regulation from top-down processes [45]. Excessive activation of bottom-up processing can increase sensitivity to external stimuli, perceiving them as threats [45–47]. Our findings contribute to a better understanding of the intricate relationship between altered FC and clinical symptoms of MDD. Additionally, we shed light on the potential impact of AOO on this relationship, underscoring the necessity for further research on the AOO effect.

In our study, we did not find a significant main effect of age on the components of intra-networks and inter-networks, which contrasts with findings from previous studies. Yong He et al. utilized 33,809 resting-state fMRI scans from individuals aged 32 postmenstrual weeks to 80 years old to construct a lifespan curve of functional connections [48]. This curve showed a nonlinear increase in functional connectivity starting from 32 postmenstrual weeks, peaking at 40.0 years, with the highest growth rate occurring at 18 years [48]. However, in our study, we did not observe a main effect of age, possibly due to limitations in sample size.

Additionally, sensitivity analysis revealed that when duration and the number of episodes were included as covariates, the results of intra-network FC within the DAN and LN remained unchanged after FDR correction. Regarding inter-network FC, we observed a main effect of diagnosis on the intra-network FC of VN and LN before FDR correction, with a consistent trend of increased FC in the MDD group. This indicates that our findings were not significantly affected by the duration and number of episodes.

However, our study has several limitations. Firstly, it is a cross-sectional study. Longitudinal studies are necessary to investigate the effects of depression onset age on neurodevelopmental trajectories and determine whether the identified abnormal functional connectivity is present in high-risk healthy individuals. Secondly, the number of participants, particularly healthy adolescents, was limited. This might explain why the main effect of age was not observed. A future large-sample, multi-site study is essential to validate our findings. Thirdly, we did not include the subcortical networks of Tian [49] in this study due to the inability to achieve surface-based registration for subcortical networks. Fourthly, the inclusion criteria for adult-onset MDD patients did not restrict the number of episodes or duration of illness. Although sensitivity analysis was conducted to assess whether the FC difference was due to the duration and number of episodes, it would be preferable to focus on first-episode patients.

In conclusion, our study highlights shared and distinct alterations in large-scale brain networks among both adolescent-onset and adult-onset MDD. Regarding shared alterations, we found increased FC in the VN, LN, VN-DAN, VN-LN, and LN-DMN networks in both groups. In terms of unique alterations, adolescent-onset patients exhibited significantly lower DAN connectivity compared to adolescent HCs, while adult-onset patients showed significantly higher DAN connectivity compared to adult HCs. Furthermore, the increased FC alteration in the LN network was specific to adult-onset MDD patients. These findings enhance our understanding of the neuroimaging mechanisms involved in adolescent-onset and adult-onset MDD, offering insights for clinical subtyping and treatment approaches. Additionally, our results emphasize the importance of considering age when investigating biomarkers for MDD.

### Supplementary information

supplymentary materials

## Data Availability

Data is available on request from the corresponding author.
