# Supplementary material for: Shared and unique alterations of large-scale network connectivity in drug-free adolescent-onset and adult-onset major depressive disorder
Source: Transl Psychiatry. 2024 Jun 12;14:255. doi: 10.1038/s41398-024-02974-0 (PMC11169372; doi:10.1038/s41398-024-02974-0)
Supplement: Supplementary file 1 — supplymentary materials [file 41398_2024_2974_MOESM1_ESM.docx]

**Supplemental Information**

**The composition of each HAMD sub-score**

The composition of HAMD sub-scores includes five dimensions: anxiety/somatization; weight; cognitive impairment; retardation and sleep disturbance. The anxiety/ somatisation (18 points) consists of (1) psychic anxiety; (2) somatic anxiety; (3) gastro-intestinal symptoms; (4) general somatic symptoms; (5)hypochondriasis; and (6) insight. The weight (2 points)refers to loss of weight. The cognitive impairment (12 points) consists of (1) feelings of guilt; (2) suicide; and (3)agitated behavior. The retardation dimension (14 points) was composed of: (1) depressed mood; (2) loss ofinterest in activity, hobbies or work; (3) slowness of thought and speech, impairedability to concentrate, decreased motor activity; and (4) sexual symptoms. The sleep disturbance (6 points) consists of (1) difficulty in falling asleep; (2) light sleep; and (3) early awakening.

**The main effects of diagnosis, age, duration, the number of episodes, and their interactive impact on FC**

1. Intra-network Connectivity Analysis

We identified a diagnosis main effect on the intra-network FC of VN and LN (F =6.374, pFDR = 0.044; F = 12.113, pFDR = 0.005), indicating that these FC abnormalities were unrelated to age stratification. Post-hoc analysis revealed shared increased network connectivity in the MDD group. Furthermore, we found an interaction effect between diagnosis and age on the intra-network FC within the VN, DAN and LN (F = 6.452, pFDR = 0.028; F = 10.851, pFDR = 0.009; F = 8.219, pFDR = 0.017). Post-hoc analysis demonstrated that intra-network connectivity of VN, DAN and LN (p = 0.002, p = 0.008, p < 0.001, Bonferroni-corrected) in adult-onset MDD patients was significantly higher than that in adult HCs, whereas DAN connectivity in adolescent-onset MDD patients was significantly lower than that in the adolescent HCs (p = 0.032, Bonferroni-corrected). And intra-network connectivity of VN in adult-onset MDD patients was significantly higher than that in adolescent-onset MDD patients (p = 0.019, Bonferroni-corrected). Additionally, intra-network connectivity of DAN and LN in adolescent HCs was significantly higher than that in the adult HCs (p = 0.002, p = 0.011, Bonferroni-corrected). No significant differences were found between the components of other intra-networks. No significant age main effect was identified among the components of intra-networks.

(2)Inter-network Connectivity Analysis

We observed a diagnosis main effect on the inter-network FC of VN-SMN, VN-DAN, VN-LN, VN-DMN and LN-DMN (F = 5.658, p = 0.019; F = 5.189, p = 0.024; F = 6.498, p = 0.012; F = 4.748, p = 0.031; F = 8.933, p = 0.003), which were uncorrelated with age stratification. Post-hoc analysis revealed shared increased network connectivity in the MDD group. No significant interaction effect or main effect was found between the connectivity of other networks after FDR correction. No significant interaction effect was found between the connectivity of other networks. No significant age main effect was identified between the connectivity of networks.

**Supplemental Figure**

**Fig. S1:** The averaged functional connectivity matrix among 400 ROIs of the whole brain in adolescents with MD, adults with MDD, adolescent HCs, adult HCs. The color bar indicates the strength of functional connectivity.


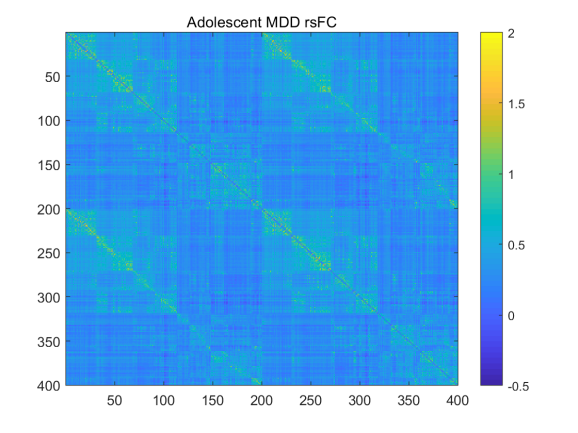

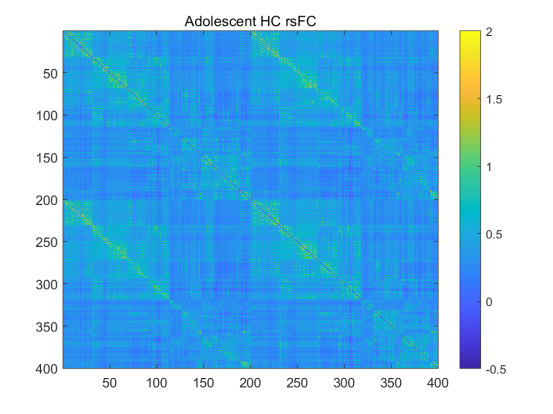


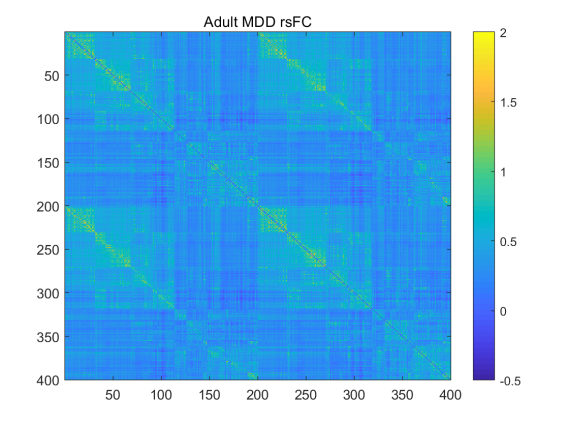

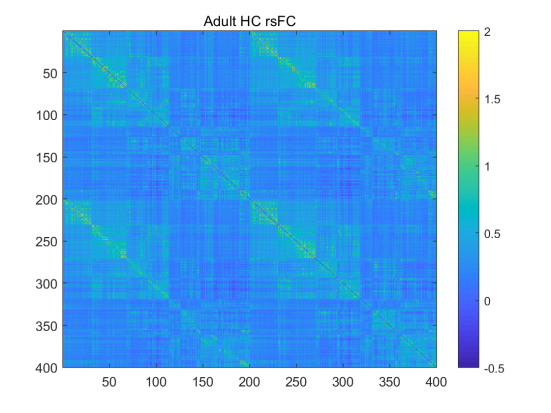


**Supplemental Table**

**Table S1 All the seed regions in the network analysis**

| **Code** | **Brain region** | **Yeo network** | **MNI coordinates** | | |
| --- | --- | --- | --- | --- | --- |
|  |  |  | **X** | **Y** | **Z** |
| 1 | Vis1_L | VN | -33 | -42 | -21 |
| 2 | Vis2_L | VN | -30 | -33 | -18 |
| 3 | Vis3_L | VN | -36 | -62 | -17 |
| 4 | Vis4_L | VN | -24 | -55 | -8 |
| 5 | Vis5_L | VN | -23 | -73 | -10 |
| 6 | Vis6_L | VN | -36 | -81 | -16 |
| 7 | Vis7_L | VN | -18 | -37 | -12 |
| 8 | Vis8_L | VN | -45 | -70 | -8 |
| 9 | Vis9_L | VN | -17 | -86 | -15 |
| 10 | Vis10_L | VN | -12 | -62 | -5 |
| 11 | Vis11_L | VN | -7 | -76 | -6 |
| 12 | Vis12_L | VN | -24 | -97 | -12 |
| 13 | Vis13_L | VN | -13 | -43 | -5 |
| 14 | Vis14_L | VN | -41 | -87 | -3 |
| 15 | Vis15_L | VN | -7 | -98 | -7 |
| 16 | Vis16_L | VN | -14 | -57 | 1 |
| 17 | Vis17_L | VN | -46 | -73 | 6 |
| 18 | Vis18_L | VN | -5 | -88 | 2 |
| 19 | Vis19_L | VN | -24 | -96 | 6 |
| 20 | Vis20_L | VN | -13 | -49 | 4 |
| 21 | Vis21_L | VN | -7 | -74 | 9 |
| 22 | Vis22_L | VN | -19 | -65 | 7 |
| 23 | Vis23_L | VN | -39 | -84 | 14 |
| 24 | Vis24_L | VN | -11 | -97 | 17 |
| 25 | Vis25_L | VN | -3 | -84 | 24 |
| 26 | Vis26_L | VN | -12 | -71 | 20 |
| 27 | Vis27_L | VN | -25 | -85 | 21 |
| 28 | Vis28_L | VN | -32 | -84 | 27 |
| 29 | Vis29_L | VN | -16 | -89 | 33 |
| 30 | Vis30_L | VN | -12 | -81 | 36 |
| 31 | Vis31_L | VN | -21 | -79 | 45 |
| 32 | SomMot1_L | SMN | -50 | -9 | 0 |
| 33 | SomMot2_L | SMN | -56 | -22 | 8 |
| 34 | SomMot3_L | SMN | -36 | -24 | 10 |
| 35 | SomMot4_L | SMN | -38 | -9 | 13 |
| 36 | SomMot5_L | SMN | -36 | -26 | 19 |
| 37 | SomMot6_L | SMN | -59 | -37 | 16 |
| 38 | SomMot7_L | SMN | -41 | -35 | 14 |
| 39 | SomMot8_L | SMN | -49 | -13 | 14 |
| 40 | SomMot9_L | SMN | -60 | -2 | 11 |
| 41 | SomMot10_L | SMN | -48 | -24 | 18 |
| 42 | SomMot11_L | SMN | -61 | -18 | 19 |
| 43 | SomMot12_L | SMN | -59 | -2 | 23 |
| 44 | SomMot13_L | SMN | -63 | -10 | 31 |
| 45 | SomMot14_L | SMN | -53 | -9 | 30 |
| 46 | SomMot15_L | SMN | -55 | -20 | 41 |
| 47 | SomMot16_L | SMN | -51 | -7 | 43 |
| 48 | SomMot17_L | SMN | -48 | -16 | 40 |
| 49 | SomMot18_L | SMN | -8 | -15 | 47 |
| 50 | SomMot19_L | SMN | -41 | -14 | 47 |
| 51 | SomMot20_L | SMN | -49 | -17 | 54 |
| 52 | SomMot21_L | SMN | -48 | -29 | 58 |
| 53 | SomMot22_L | SMN | -39 | -25 | 53 |
| 54 | SomMot23_L | SMN | -9 | -38 | 54 |
| 55 | SomMot24_L | SMN | -4 | -25 | 56 |
| 56 | SomMot25_L | SMN | -4 | -9 | 59 |
| 57 | SomMot26_L | SMN | -36 | -19 | 65 |
| 58 | SomMot27_L | SMN | -30 | -46 | 63 |
| 59 | SomMot28_L | SMN | -32 | -29 | 63 |
| 60 | SomMot29_L | SMN | -30 | -38 | 65 |
| 61 | SomMot30_L | SMN | -23 | -11 | 65 |
| 62 | SomMot31_L | SMN | -19 | -24 | 67 |
| 63 | SomMot32_L | SMN | -9 | -42 | 70 |
| 64 | SomMot33_L | SMN | -4 | -25 | 69 |
| 65 | SomMot34_L | SMN | -14 | -11 | 73 |
| 66 | SomMot35_L | SMN | -19 | -31 | 69 |
| 67 | SomMot36_L | SMN | -19 | -40 | 72 |
| 68 | SomMot37_L | SMN | -12 | -27 | 73 |
| 69 | DorsAttn-Post1_L | DAN | -45 | -42 | -21 |
| 70 | DorsAttn-Post2_L | DAN | -49 | -56 | -15 |
| 71 | DorsAttn-Post3_L | DAN | -55 | -62 | -1 |
| 72 | DorsAttn-Post4_L | DAN | -48 | -65 | 15 |
| 73 | DorsAttn-Post5_L | DAN | -26 | -70 | 31 |
| 74 | DorsAttn-Post6_L | DAN | -55 | -32 | 45 |
| 75 | DorsAttn-Post7_L | DAN | -23 | -65 | 46 |
| 76 | DorsAttn-Post8_L | DAN | -46 | -29 | 44 |
| 77 | DorsAttn-Post9_L | DAN | -33 | -46 | 41 |
| 78 | DorsAttn-Post10_L | DAN | -29 | -58 | 50 |
| 79 | DorsAttn-Post11_L | DAN | -39 | -37 | 49 |
| 80 | DorsAttn-Post12_L | DAN | -36 | -52 | 56 |
| 81 | DorsAttn-Post13_L | DAN | -15 | -71 | 57 |
| 82 | DorsAttn-Post14_L | DAN | -29 | -61 | 62 |
| 83 | DorsAttn-Post15_L | DAN | -7 | -59 | 63 |
| 84 | DorsAttn-Post16_L | DAN | -20 | -57 | 66 |
| 85 | DorsAttn-Post17_L | DAN | -13 | -50 | 72 |
| 86 | DorsAttn-FEF1_L | DAN | -40 | -3 | 51 |
| 87 | DorsAttn-FEF2_L | DAN | -25 | -1 | 55 |
| 88 | DorsAttn-FEF3_L | DAN | -30 | -8 | 52 |
| 89 | DorsAttn-FEF4_L | DAN | -21 | 5 | 65 |
| 90 | DorsAttn-PrCv1_L | DAN | -49 | 6 | 26 |
| 91 | DorsAttn-PrCv2_L | DAN | -50 | 3 | 38 |
| 92 | SalVentAttn-ParOper1_L | VAN | -55 | -32 | 22 |
| 93 | SalVentAttn-ParOper2_L | VAN | -58 | -44 | 27 |
| 94 | SalVentAttn-ParOper3_L | VAN | -61 | -23 | 33 |
| 95 | SalVentAttn-ParOper4_L | VAN | -61 | -36 | 33 |
| 96 | SalVentAttn-TempOcc1_L | VAN | -57 | -54 | 10 |
| 97 | SalVentAttn-FrOperIns1_L | VAN | -44 | 5 | -17 |
| 98 | SalVentAttn-FrOperIns2_L | VAN | -39 | 2 | -4 |
| 99 | SalVentAttn-FrOperIns3_L | VAN | -33 | 25 | -1 |
| 100 | SalVentAttn-FrOperIns4_L | VAN | -40 | -15 | -2 |
| 101 | SalVentAttn-FrOperIns5_L | VAN | -33 | 19 | 8 |
| 102 | SalVentAttn-FrOperIns6_L | VAN | -36 | 4 | 11 |
| 103 | SalVentAttn-FrOperIns7_L | VAN | -43 | 12 | 2 |
| 104 | SalVentAttn-FrOperIns8_L | VAN | -50 | 1 | 5 |
| 105 | SalVentAttn-FrOperIns9_L | VAN | -52 | 9 | 13 |
| 106 | SalVentAttn-PFCl1_L | VAN | -29 | 43 | 30 |
| 107 | SalVentAttn-Med1_L | VAN | -6 | 22 | 31 |
| 108 | SalVentAttn-Med2_L | VAN | -7 | 0 | 41 |
| 109 | SalVentAttn-Med3_L | VAN | -11 | -27 | 41 |
| 110 | SalVentAttn-Med4_L | VAN | -5 | 9 | 48 |
| 111 | SalVentAttn-Med5_L | VAN | -13 | -41 | 47 |
| 112 | SalVentAttn-Med6_L | VAN | -6 | -49 | 57 |
| 113 | SalVentAttn-Med7_L | VAN | -8 | -3 | 71 |
| 114 | Limbic-OFC1_L | LN | -12 | 25 | -21 |
| 115 | Limbic-OFC2_L | LN | -24 | 23 | -20 |
| 116 | Limbic-OFC3_L | LN | -10 | 47 | -21 |
| 117 | Limbic-OFC4_L | LN | -4 | 23 | -19 |
| 118 | Limbic-OFC5_L | LN | -15 | 65 | -8 |
| 119 | Limbic-TempPole1_L | LN | -37 | -5 | -42 |
| 120 | Limbic-TempPole2_L | LN | -25 | 6 | -39 |
| 121 | Limbic-TempPole3_L | LN | -26 | -9 | -33 |
| 122 | Limbic-TempPole4_L | LN | -54 | -21 | -31 |
| 123 | Limbic-TempPole5_L | LN | -40 | -21 | -27 |
| 124 | Limbic-TempPole6_L | LN | -44 | 13 | -34 |
| 125 | Limbic-TempPole7_L | LN | -32 | 12 | -29 |
| 126 | Limbic-TempPole8_L | LN | -21 | -21 | -26 |
| 127 | Cont-Par1_L | FPN | -29 | -74 | 42 |
| 128 | Cont-Par2_L | FPN | -58 | -42 | 45 |
| 129 | Cont-Par3_L | FPN | -53 | -50 | 45 |
| 130 | Cont-Par4_L | FPN | -35 | -62 | 48 |
| 131 | Cont-Par5_L | FPN | -42 | -52 | 49 |
| 132 | Cont-Par6_L | FPN | -45 | -41 | 47 |
| 133 | Cont-Temp1_L | FPN | -60 | -49 | -10 |
| 134 | Cont-OFC1_L | FPN | -27 | 49 | -14 |
| 135 | Cont-PFCl1_L | FPN | -42 | 49 | -6 |
| 136 | Cont-PFCl2_L | FPN | -48 | 35 | 10 |
| 137 | Cont-PFCl3_L | FPN | -38 | 49 | 11 |
| 138 | Cont-PFCl4_L | FPN | -28 | 57 | 13 |
| 139 | Cont-PFCl5_L | FPN | -42 | 38 | 22 |
| 140 | Cont-PFCl6_L | FPN | -45 | 20 | 27 |
| 141 | Cont-PFCl7_L | FPN | -39 | 7 | 34 |
| 142 | Cont-PFCl8_L | FPN | -36 | 32 | 38 |
| 143 | Cont-PFCv1_L | FPN | -34 | 16 | -8 |
| 144 | Cont-pCun1_L | FPN | -9 | -77 | 45 |
| 145 | Cont-pCun2_L | FPN | -5 | -64 | 52 |
| 146 | Cont-Cing1_L | FPN | -3 | 5 | 29 |
| 147 | Cont-Cing2_L | FPN | -4 | -22 | 29 |
| 148 | Cont-PFCmp1_L | FPN | -4 | 28 | 47 |
| 149 | Default-Temp1_L | DMN | -54 | -2 | -30 |
| 150 | Default-Temp2_L | DMN | -62 | -18 | -21 |
| 151 | Default-Temp3_L | DMN | -60 | -36 | -18 |
| 152 | Default-Temp4_L | DMN | -57 | -9 | -14 |
| 153 | Default-Temp5_L | DMN | -53 | 6 | -11 |
| 154 | Default-Temp6_L | DMN | -61 | -35 | -3 |
| 155 | Default-Temp7_L | DMN | -52 | -22 | -6 |
| 156 | Default-Temp8_L | DMN | -61 | -13 | -3 |
| 157 | Default-Temp9_L | DMN | -62 | -32 | 5 |
| 158 | Default-Temp10_L | DMN | -52 | -43 | 5 |
| 159 | Default-Par1_L | DMN | -59 | -49 | 16 |
| 160 | Default-Par2_L | DMN | -45 | -58 | 21 |
| 161 | Default-Par3_L | DMN | -40 | -79 | 30 |
| 162 | Default-Par4_L | DMN | -47 | -64 | 31 |
| 163 | Default-Par5_L | DMN | -57 | -55 | 30 |
| 164 | Default-Par6_L | DMN | -41 | -72 | 43 |
| 165 | Default-Par7_L | DMN | -49 | -60 | 47 |
| 166 | Default-PFC1_L | DMN | -36 | 22 | -16 |
| 167 | Default-PFC2_L | DMN | -36 | 37 | -13 |
| 168 | Default-PFC3_L | DMN | -5 | 55 | -10 |
| 169 | Default-PFC4_L | DMN | -6 | 35 | -9 |
| 170 | Default-PFC5_L | DMN | -46 | 32 | -10 |
| 171 | Default-PFC6_L | DMN | -28 | 58 | -1 |
| 172 | Default-PFC7_L | DMN | -48 | 28 | 0 |
| 173 | Default-PFC8_L | DMN | -6 | 59 | 7 |
| 174 | Default-PFC9_L | DMN | -6 | 45 | 6 |
| 175 | Default-PFC10_L | DMN | -53 | 19 | 11 |
| 176 | Default-PFC11_L | DMN | -16 | 67 | 8 |
| 177 | Default-PFC12_L | DMN | -5 | 34 | 21 |
| 178 | Default-PFC13_L | DMN | -4 | 51 | 28 |
| 179 | Default-PFC14_L | DMN | -14 | 58 | 31 |
| 180 | Default-PFC15_L | DMN | -22 | 51 | 31 |
| 181 | Default-PFC16_L | DMN | -25 | 28 | 43 |
| 182 | Default-PFC17_L | DMN | -41 | 19 | 48 |
| 183 | Default-PFC18_L | DMN | -8 | 43 | 51 |
| 184 | Default-PFC19_L | DMN | -18 | 36 | 48 |
| 185 | Default-PFC20_L | DMN | -42 | 7 | 48 |
| 186 | Default-PFC21_L | DMN | -30 | 14 | 57 |
| 187 | Default-PFC22_L | DMN | -22 | 20 | 52 |
| 188 | Default-PFC23_L | DMN | -13 | 24 | 61 |
| 189 | Default-PFC24_L | DMN | -6 | 10 | 65 |
| 190 | Default-pCunPCC1_L | DMN | -8 | -52 | 9 |
| 191 | Default-pCunPCC2_L | DMN | -13 | -61 | 19 |
| 192 | Default-pCunPCC3_L | DMN | -4 | -53 | 20 |
| 193 | Default-pCunPCC4_L | DMN | -6 | -41 | 24 |
| 194 | Default-pCunPCC5_L | DMN | -10 | -70 | 32 |
| 195 | Default-pCunPCC6_L | DMN | -5 | -60 | 30 |
| 196 | Default-pCunPCC7_L | DMN | -7 | -44 | 32 |
| 197 | Default-pCunPCC8_L | DMN | -4 | -34 | 38 |
| 198 | Default-pCunPCC9_L | DMN | -3 | -15 | 37 |
| 199 | Default-pCunPCC10_L | DMN | -3 | -68 | 41 |
| 200 | Default-pCunPCC11_L | DMN | -7 | -51 | 43 |
| 201 | Vis1_R | VN | 34 | -37 | -23 |
| 202 | Vis2_R | VN | 31 | -31 | -18 |
| 203 | Vis3_R | VN | 36 | -53 | -17 |
| 204 | Vis4_R | VN | 37 | -73 | -16 |
| 205 | Vis5_R | VN | 26 | -52 | -9 |
| 206 | Vis6_R | VN | 23 | -74 | -11 |
| 207 | Vis7_R | VN | 18 | -36 | -12 |
| 208 | Vis8_R | VN | 20 | -88 | -13 |
| 209 | Vis9_R | VN | 50 | -64 | -9 |
| 210 | Vis10_R | VN | 42 | -84 | -12 |
| 211 | Vis11_R | VN | 9 | -72 | -5 |
| 212 | Vis12_R | VN | 13 | -58 | -3 |
| 213 | Vis13_R | VN | 25 | -97 | -10 |
| 214 | Vis14_R | VN | 18 | -45 | -3 |
| 215 | Vis15_R | VN | 8 | -92 | -2 |
| 216 | Vis16_R | VN | 48 | -66 | 4 |
| 217 | Vis17_R | VN | 14 | -46 | 4 |
| 218 | Vis18_R | VN | 35 | -89 | 2 |
| 219 | Vis19_R | VN | 9 | -74 | 9 |
| 220 | Vis20_R | VN | 22 | -59 | 6 |
| 221 | Vis21_R | VN | 24 | -99 | 7 |
| 222 | Vis22_R | VN | 43 | -79 | 10 |
| 223 | Vis23_R | VN | 13 | -94 | 19 |
| 224 | Vis24_R | VN | 16 | -66 | 19 |
| 225 | Vis25_R | VN | 5 | -80 | 24 |
| 226 | Vis26_R | VN | 27 | -87 | 21 |
| 227 | Vis27_R | VN | 36 | -79 | 24 |
| 228 | Vis28_R | VN | 14 | -78 | 34 |
| 229 | Vis29_R | VN | 16 | -87 | 36 |
| 230 | Vis30_R | VN | 29 | -78 | 37 |
| 231 | SomMot1_R | VN | 53 | 3 | -6 |
| 232 | SomMot2_R | SMN | 62 | -19 | 0 |
| 233 | SomMot3_R | SMN | 53 | -14 | 6 |
| 234 | SomMot4_R | SMN | 39 | -19 | 5 |
| 235 | SomMot5_R | SMN | 37 | -8 | 14 |
| 236 | SomMot6_R | SMN | 35 | -21 | 14 |
| 237 | SomMot7_R | SMN | 60 | -24 | 11 |
| 238 | SomMot8_R | SMN | 65 | -34 | 11 |
| 239 | SomMot9_R | SMN | 41 | -13 | 18 |
| 240 | SomMot10_R | SMN | 41 | -29 | 18 |
| 241 | SomMot11_R | SMN | 50 | -10 | 13 |
| 242 | SomMot12_R | SMN | 59 | 1 | 10 |
| 243 | SomMot13_R | SMN | 49 | -21 | 19 |
| 244 | SomMot14_R | SMN | 62 | -12 | 15 |
| 245 | SomMot15_R | SMN | 61 | 6 | 30 |
| 246 | SomMot16_R | SMN | 60 | -5 | 27 |
| 247 | SomMot17_R | SMN | 61 | -14 | 30 |
| 248 | SomMot18_R | SMN | 52 | -6 | 37 |
| 249 | SomMot19_R | SMN | 54 | -17 | 40 |
| 250 | SomMot20_R | SMN | 11 | -17 | 41 |
| 251 | SomMot21_R | SMN | 52 | -13 | 49 |
| 252 | SomMot22_R | SMN | 44 | -10 | 49 |
| 253 | SomMot23_R | SMN | 49 | -26 | 56 |
| 254 | SomMot24_R | SMN | 7 | -10 | 51 |
| 255 | SomMot25_R | SMN | 43 | -21 | 54 |
| 256 | SomMot26_R | SMN | 37 | -20 | 64 |
| 257 | SomMot27_R | SMN | 32 | -34 | 63 |
| 258 | SomMot28_R | SMN | 31 | -41 | 64 |
| 259 | SomMot29_R | SMN | 34 | -27 | 61 |
| 260 | SomMot30_R | SMN | 4 | -25 | 58 |
| 261 | SomMot31_R | SMN | 29 | -11 | 65 |
| 262 | SomMot32_R | SMN | 9 | -40 | 68 |
| 263 | SomMot33_R | SMN | 21 | -24 | 67 |
| 264 | SomMot34_R | SMN | 22 | -29 | 68 |
| 265 | SomMot35_R | SMN | 22 | -35 | 71 |
| 266 | SomMot36_R | SMN | 17 | -6 | 69 |
| 267 | SomMot37_R | SMN | 16 | -47 | 74 |
| 268 | SomMot38_R | SMN | 5 | -22 | 72 |
| 269 | SomMot39_R | DAN | 17 | -18 | 73 |
| 270 | SomMot40_R | DAN | 13 | -33 | 76 |
| 271 | DorsAttn-Post1_R | DAN | 50 | -49 | -18 |
| 272 | DorsAttn-Post2_R | DAN | 59 | -55 | -2 |
| 273 | DorsAttn-Post3_R | DAN | 54 | -56 | 12 |
| 274 | DorsAttn-Post4_R | DAN | 45 | -75 | 31 |
| 275 | DorsAttn-Post5_R | DAN | 32 | -66 | 35 |
| 276 | DorsAttn-Post6_R | DAN | 57 | -23 | 44 |
| 277 | DorsAttn-Post7_R | DAN | 19 | -79 | 50 |
| 278 | DorsAttn-Post8_R | DAN | 44 | -37 | 50 |
| 279 | DorsAttn-Post9_R | DAN | 45 | -28 | 42 |
| 280 | DorsAttn-Post10_R | DAN | 31 | -64 | 53 |
| 281 | DorsAttn-Post11_R | DAN | 36 | -44 | 45 |
| 282 | DorsAttn-Post12_R | DAN | 21 | -69 | 53 |
| 283 | DorsAttn-Post13_R | DAN | 35 | -36 | 51 |
| 284 | DorsAttn-Post14_R | DAN | 34 | -50 | 54 |
| 285 | DorsAttn-Post15_R | DAN | 8 | -71 | 53 |
| 286 | DorsAttn-Post16_R | DAN | 27 | -58 | 61 |
| 287 | DorsAttn-Post17_R | DAN | 7 | -54 | 59 |
| 288 | DorsAttn-Post18_R | DAN | 14 | -64 | 65 |
| 289 | DorsAttn-Post19_R | DAN | 24 | -50 | 68 |
| 290 | DorsAttn-FEF1_R | DAN | 39 | -3 | 53 |
| 291 | DorsAttn-FEF2_R | DAN | 27 | -3 | 52 |
| 292 | DorsAttn-FEF3_R | VAN | 25 | -3 | 64 |
| 293 | DorsAttn-PrCv1_R | VAN | 49 | 8 | 25 |
| 294 | SalVentAttn-TempOccPar1_R | VAN | 59 | -46 | 7 |
| 295 | SalVentAttn-TempOccPar2_R | VAN | 51 | -41 | 13 |
| 296 | SalVentAttn-TempOccPar3_R | VAN | 62 | -40 | 22 |
| 297 | SalVentAttn-TempOccPar4_R | VAN | 58 | -31 | 24 |
| 298 | SalVentAttn-TempOccPar5_R | VAN | 60 | -22 | 22 |
| 299 | SalVentAttn-TempOccPar6_R | VAN | 63 | -26 | 38 |
| 300 | SalVentAttn-TempOccPar7_R | VAN | 62 | -37 | 37 |
| 301 | SalVentAttn-PrC1_R | VAN | 51 | 3 | 41 |
| 302 | SalVentAttn-FrOperIns1_R | VAN | 40 | 5 | -15 |
| 303 | SalVentAttn-FrOperIns2_R | VAN | 41 | 8 | -3 |
| 304 | SalVentAttn-FrOperIns3_R | VAN | 40 | -10 | -4 |
| 305 | SalVentAttn-FrOperIns4_R | VAN | 39 | -2 | 6 |
| 306 | SalVentAttn-FrOperIns5_R | VAN | 37 | 23 | 5 |
| 307 | SalVentAttn-FrOperIns6_R | VAN | 38 | 7 | 11 |
| 308 | SalVentAttn-FrOperIns7_R | VAN | 49 | 5 | 3 |
| 309 | SalVentAttn-FrOperIns8_R | VAN | 54 | 12 | 12 |
| 310 | SalVentAttn-PFCl1_R | VAN | 33 | 45 | 28 |
| 311 | SalVentAttn-Med1_R | VAN | 7 | 19 | 35 |
| 312 | SalVentAttn-Med2_R | VAN | 7 | 2 | 43 |
| 313 | SalVentAttn-Med3_R | VAN | 12 | -34 | 43 |
| 314 | SalVentAttn-Med4_R | LN | 6 | 11 | 58 |
| 315 | SalVentAttn-Med5_R | LN | 10 | -43 | 53 |
| 316 | SalVentAttn-Med6_R | LN | 11 | -32 | 50 |
| 317 | SalVentAttn-Med7_R | LN | 7 | -2 | 67 |
| 318 | SalVentAttn-Med8_R | LN | 16 | 7 | 69 |
| 319 | Limbic-OFC1_R | LN | 13 | 24 | -21 |
| 320 | Limbic-OFC2_R | LN | 23 | 22 | -21 |
| 321 | Limbic-OFC3_R | LN | 8 | 47 | -23 |
| 322 | Limbic-OFC4_R | LN | 20 | 43 | -18 |
| 323 | Limbic-OFC5_R | LN | 5 | 22 | -21 |
| 324 | Limbic-OFC6_R | LN | 9 | 63 | -14 |
| 325 | Limbic-TempPole1_R | LN | 28 | -1 | -40 |
| 326 | Limbic-TempPole2_R | LN | 49 | -7 | -39 |
| 327 | Limbic-TempPole3_R | FPN | 37 | 17 | -38 |
| 328 | Limbic-TempPole4_R | FPN | 39 | -15 | -31 |
| 329 | Limbic-TempPole5_R | FPN | 29 | 12 | -30 |
| 330 | Limbic-TempPole6_R | FPN | 50 | -28 | -26 |
| 331 | Limbic-TempPole7_R | FPN | 23 | -18 | -27 |
| 332 | Cont-Par1_R | FPN | 54 | -53 | 44 |
| 333 | Cont-Par2_R | FPN | 56 | -41 | 48 |
| 334 | Cont-Par3_R | FPN | 35 | -71 | 47 |
| 335 | Cont-Par4_R | FPN | 54 | -33 | 51 |
| 336 | Cont-Par5_R | FPN | 47 | -44 | 46 |
| 337 | Cont-Par6_R | FPN | 41 | -55 | 48 |
| 338 | Cont-Temp1_R | FPN | 62 | -28 | -20 |
| 339 | Cont-Temp2_R | FPN | 63 | -42 | -11 |
| 340 | Cont-PFCv1_R | FPN | 34 | 21 | -8 |
| 341 | Cont-PFCl1_R | FPN | 28 | 55 | -14 |
| 342 | Cont-PFCl2_R | FPN | 42 | 51 | -6 |
| 343 | Cont-PFCl3_R | FPN | 27 | 59 | 3 |
| 344 | Cont-PFCl4_R | FPN | 49 | 40 | 5 |
| 345 | Cont-PFCl5_R | FPN | 42 | 46 | 14 |
| 346 | Cont-PFCl6_R | FPN | 50 | 30 | 18 |
| 347 | Cont-PFCl7_R | FPN | 48 | 18 | 23 |
| 348 | Cont-PFCl8_R | FPN | 25 | 54 | 25 |
| 349 | Cont-PFCl9_R | DMN | 47 | 29 | 28 |
| 350 | Cont-PFCl10_R | DMN | 39 | 11 | 34 |
| 351 | Cont-PFCl11_R | DMN | 39 | 33 | 38 |
| 352 | Cont-PFCl12_R | DMN | 45 | 19 | 44 |
| 353 | Cont-PFCl13_R | DMN | 43 | 7 | 51 |
| 354 | Cont-PFCl14_R | DMN | 34 | 15 | 56 |
| 355 | Cont-PFCl15_R | DMN | 24 | 10 | 58 |
| 356 | Cont-pCun1_R | DMN | 13 | -71 | 39 |
| 357 | Cont-pCun2_R | DMN | 5 | -64 | 44 |
| 358 | Cont-Cing1_R | DMN | 6 | -26 | 28 |
| 359 | Cont-Cing2_R | DMN | 5 | 1 | 30 |
| 360 | Cont-PFCmp1_R | DMN | 8 | 35 | 25 |
| 361 | Cont-PFCmp2_R | DMN | 5 | 28 | 48 |
| 362 | Default-Par1_R | DMN | 55 | -46 | 19 |
| 363 | Default-Par2_R | DMN | 48 | -64 | 22 |
| 364 | Default-Par3_R | DMN | 53 | -53 | 26 |
| 365 | Default-Par4_R | DMN | 55 | -45 | 33 |
| 366 | Default-Par5_R | DMN | 47 | -64 | 42 |
| 367 | Default-Temp1_R | DMN | 49 | 9 | -33 |
| 368 | Default-Temp2_R | DMN | 61 | -8 | -23 |
| 369 | Default-Temp3_R | DMN | 47 | 16 | -20 |
| 370 | Default-Temp4_R | DMN | 55 | -4 | -14 |
| 371 | Default-Temp5_R | DMN | 63 | -23 | -7 |
| 372 | Default-Temp6_R | DMN | 49 | -20 | -8 |
| 373 | Default-Temp7_R | DMN | 63 | -38 | 0 |
| 374 | Default-Temp8_R | DMN | 50 | -33 | 2 |
| 375 | Default-PFCv1_R | DMN | 35 | 23 | -18 |
| 376 | Default-PFCv2_R | DMN | 35 | 38 | -13 |
| 377 | Default-PFCv3_R | DMN | 48 | 32 | -8 |
| 378 | Default-PFCv4_R | DMN | 54 | 24 | 6 |
| 379 | Default-PFCdPFCm1_R | DMN | 5 | 41 | -11 |
| 380 | Default-PFCdPFCm2_R | DMN | 9 | 67 | 1 |
| 381 | Default-PFCdPFCm3_R | DMN | 7 | 42 | 4 |
| 382 | Default-PFCdPFCm4_R | DMN | 7 | 54 | 13 |
| 383 | Default-PFCdPFCm5_R | DMN | 17 | 65 | 16 |
| 384 | Default-PFCdPFCm6_R | DMN | 6 | 25 | 18 |
| 385 | Default-PFCdPFCm7_R | DMN | 6 | 58 | 29 |
| 386 | Default-PFCdPFCm8_R | DMN | 16 | 52 | 36 |
| 387 | Default-PFCdPFCm9_R | DMN | 5 | 44 | 40 |
| 388 | Default-PFCdPFCm10_R | DMN | 26 | 34 | 39 |
| 389 | Default-PFCdPFCm11_R | DMN | 14 | 39 | 52 |
| 390 | Default-PFCdPFCm12_R | DMN | 24 | 26 | 51 |
| 391 | Default-PFCdPFCm13_R | DMN | 12 | 20 | 63 |
| 392 | Default-pCunPCC1_R | DMN | 12 | -55 | 15 |
| 393 | Default-pCunPCC2_R | DMN | 7 | -44 | 20 |
| 394 | Default-pCunPCC3_R | DMN | 17 | -63 | 28 |
| 395 | Default-pCunPCC4_R | DMN | 6 | -52 | 23 |
| 396 | Default-pCunPCC5_R | DMN | 5 | -63 | 31 |
| 397 | Default-pCunPCC6_R | DMN | 7 | -39 | 35 |
| 398 | Default-pCunPCC7_R | DMN | 4 | -20 | 37 |
| 399 | Default-pCunPCC8_R | DMN | 10 | -53 | 35 |
| 400 | Default-pCunPCC9_R | DMN | 7 | -50 | 45 |

Notes: VN: visual network; SMN: somatosensory-motor network; DAN: dorsal attention network; VAN: ventral attention network; LN: limbic network; FPN: frontoparietal network; DMN: default mode network.
